# Replication Study in a Japanese Population to Evaluate the Association between 10 SNP Loci, Identified in European Genome-Wide Association Studies, and Type 2 Diabetes

**DOI:** 10.1371/journal.pone.0126363

**Published:** 2015-05-07

**Authors:** Ren Matsuba, Kensuke Sakai, Minako Imamura, Yasushi Tanaka, Minoru Iwata, Hiroshi Hirose, Kohei Kaku, Hiroshi Maegawa, Hirotaka Watada, Kazuyuki Tobe, Atsunori Kashiwagi, Ryuzo Kawamori, Shiro Maeda

**Affiliations:** 1 Laboratory for Endocrinology, Metabolism and Kidney Diseases, RIKEN Center for Integrative Medical Sciences, Yokohama, Japan; 2 Department of Internal Medicine, Division of Metabolism and Endocrinology, St. Marianna University School of Medicine, Kawasaki, Japan; 3 First Department of Internal Medicine, University of Toyama, Toyama, Japan; 4 Community Medical Support Unit, Faculty of Medicine, University of Toyama, Toyama, Japan; 5 Health Center, Keio University School of Medicine, Tokyo, Japan; 6 Department of Internal Medicine, Kawasaki Medical School, Kurashiki, Japan; 7 Department of Medicine, Shiga University of Medical Science, Otsu, Japan; 8 Department of Metabolism and Endocrinology, Juntendo University Graduate School of Medicine, Tokyo, Japan; 9 Sportology Center, Juntendo University Graduate School of Medicine, Tokyo, Japan; 10 Department of Advanced Genomic and Laboratory Medicine, Graduate School of Medicine, University of the Ryukyus, Nishihara, Japan; National Cancer Institute, National Institutes of Health, UNITED STATES

## Abstract

**Aim:**

We performed a replication study in a Japanese population to evaluate the association between type 2 diabetes and 7 susceptibility loci originally identified by European genome-wide association study (GWAS) in 2012: *ZMIZ1*, *KLHDC5*, *TLE1*, *ANKRD55*, *CILP2*, *MC4R*, and *BCAR1*. We also examined the association of 3 additional loci: *CCND2* and *GIPR*, identified in sex-differentiated analyses, and *LAMA1*, which was shown to be associated with non-obese European type 2 diabetes.

**Methods:**

We genotyped 6,972 Japanese participants (4,280 type 2 diabetes patients and 2,692 controls) for each of the 10 single nucleotide polymorphisms (SNPs): rs12571751 in *ZMIZ1*, rs10842994 near *KLHDC5*, rs2796441 near *TLE1*, rs459193 near *ANKRD55*, rs10401969 in *CILP2*, rs12970134 near *MC4R*, rs7202877 near *BCAR1*, rs11063069 near *CCND2*, rs8108269 near *GIPR*, and rs8090011 in *LAMA1* using a multiplex polymerase chain reaction invader assay. The association of each SNP locus with the disease was evaluated using a logistic regression analysis.

**Results:**

All SNPs examined in this study had the same direction of effect (odds ratio > 1.0, p = 9.77 × 10^-4^, binomial test), as in the original reports. Among them, rs12571751 in *ZMIZ1* was significantly associated with type 2 diabetes [p = 0.0041, odds ratio = 1.123, 95% confidence interval 1.037–1.215, adjusted for sex, age and body mass index (BMI)], but we did not observe significant association of the remaining 9 SNP loci with type 2 diabetes in the present Japanese population (p ≥ 0.005). A genetic risk score, constructed from the sum of risk alleles for the 7 SNP loci identified by un-stratified analyses in the European GWAS meta-analysis were associated with type 2 diabetes in the present Japanese population (p = 2.3 × 10^-4^, adjusted for sex, age and BMI).

**Conclusions:**

*ZMIZ1* locus has a significant effect on conferring susceptibility to type 2 diabetes also in the Japanese population.

## Introduction

Genetic susceptibility plays an important role in the development and/or progression of type 2 diabetes. Genome-wide association studies (GWAS) for type 2 diabetes have been extensively conducted worldwide, and over 80 susceptibility loci have been identified [1–19]. The association between susceptibility loci for type 2 diabetes identified in European GWAS and the disease have been evaluated in the Japanese population, and many European GWAS-derived loci were found to be associated with type 2 diabetes in Japanese populations, whereas others were not [8,11,18,20–29]

In 2012, a European GWAS meta-analysis identified 8 novel loci: rs12571751 in the zinc finger miz-domain containing 1 gene (*ZMIZ1*), rs10842994 near the kelch domain containing 5 gene (*KLHDC5*), rs2796441 near the transducin-like enhancer of split 1 gene (*TLE1*), rs459193 near the ankyrin repeat domain-containing protein 55 gene (*ANKRD55*), rs10401969 in the cartilage intermediate layer protein 2 gene (*CILP2*), rs12970134 near the melancortin 4 receptor gene (*MC4R*), rs7202877 near the breast cancer antiestrogen resistance 1 gene (*BCAR1*), rs516946 in the ankyrin 1 (*ANK1)* [16]. Subsequently, in a sex-differentiated analysis followed by a meta-analysis, rs11063069 near the cyclin D2 gene (*CCND2*) and rs8108269 near the gastric inhibitory polypeptide receptor gene (*GIPR*) were shown to be associated with type 2 diabetes with a genome-wide significance level, and the effect of *CCND2* locus was stronger in male, whereas the association of the *GIPR* locus was more significant in female [16]. Moreover, rs8090011 in the laminin alpha-1 gene (*LAMA1*) has been shown to be associated with non-obese European type 2 diabetes [15]. Among the 11 loci, an independent Japanese GWAS identified *ANK1* as a susceptibility locus for type 2 diabetes [17]. However, the remaining 10 SNP loci have not been well evaluated in the Japanese population, although the association of 4 SNP loci (rs12571751, rs2796441, rs4591937 and rs7202877) with type 2 diabetes has been suggested [18].

In this study, we examined the association of these 10 susceptibility loci, originally identified in European GWAS, with type 2 diabetes in a Japanese population.

## Materials and Methods

### Participants and DNA Preparation

We enrolled 4,280 type 2 diabetes patients who regularly visited the outpatient clinics of the Shiga University of Medical Science, Kawasaki Medical School, St. Marianna University, Juntendo University, and the University of Toyama or who were registered in BioBank Japan [11]. Diabetes mellitus was diagnosed according to the World Health Organization (WHO) criteria [30], and type 2 diabetes was clinically defined as a gradual adult-onset diabetes. Patients who tested positive for antibodies to glutamic acid decarboxylase or who were diagnosed with a monogenic form of the disease, such as mitochondrial disease or maturity-onset diabetes of the young, were excluded from the present study. We also recruited 2,692 controls who underwent annual health check-ups at Keio University, St. Marianna University, or Toyama University Hospital or from the general Japanese population registered in the Japanese single nucleotide polymorphism (SNP) database [11]. Peripheral blood samples for genomic DNA were extracted using the standard phenol-chloroform procedure. Obesity was defined as body mass index (BMI) ≥ 25 kg/m^2^ according to the criteria of the Japan Society for the Study of Obesity [31].

### Ethics Statements

All participants agreed to the protocol of this study and provided written informed consent. The study protocol was approved by the ethics committees of RIKEN Yokohama Institutes and each of the participating institutes: Shiga University of Medical Science, Kawasaki Medical School, St. Marianna University, Juntendo University, the University of Toyama, and Keio University.

### SNP Genotyping

We selected 10 autosomal SNPs identified by European GWAS in 2012, including, rs12571751 in *ZMIZ1*, rs10842994 near *KLHDC5*, rs2796441 near *TLE1*, rs459193 near *ANKRD55*, rs10401969 in *CILP2*, rs12970134 near *MC4R*, rs7202877 near *BCAR1*, rs11063069 near *CCND2*, rs8108269 near *GIPR* [16], and rs8090011 in *LAMA1* [15].

Genotyping was performed using the multiplex-polymerase chain reaction (PCR) invader assay as reported previously [32]. The genotyping success rates for all of the SNPs were over 95.8% (S1 Table). The genotype concordance rates in the 64 duplicated samples were 100%. We did not observe any significant correlation among any SNP combinations in terms of calculated regression co-efficient (r square) in this analysis.

### Statistical Analysis

We performed Hardy-Weinberg equilibrium (HWE) tests according to the method described by Nielsen et al. [33]. Differences in the genotype distribution of each SNP between cases and controls were evaluated by a logistic regression analysis with or without adjustment for age, sex, and BMI. The association of each SNP with quantitative traits, fasting plasma glucose (FPG), BMI, the homeostasis model assessment of beta-cell function (HOMA-β), and the HOMA of insulin resistance (HOMA-IR) [34,35] was evaluated by multiple linear regression analysis. Because the values of these traits in the present Japanese population showed a skewed distribution, we used log-transformed values for the analyses. Genotypes of each SNP were scored using an additive model (0, 1, and 2 for the homozygous of non-effect allele, heterozygous, and homozygous of effect allele, respectively). Statistical analyses were performed using StatView software (SAS Institute, Cary, NC, USA).

Significance was determined by Bonferroni’s method for correcting multiple testing errors, and p < 0.005 (0.05 divided by 10) was considered statistically significant.

## Results

Clinical characteristics of the participants are shown in Table 1. The genotype distributions of all SNPs were in accordance with the Hardy-Weinberg equilibrium proportions, except for rs12571751 in type 2 diabetes cases (Table 2).

**Table 1:** Clinical characteristic of participants.

	Sample size (case/control)	Type 2 diabetes	Controls	*p* value
n		4,280	2,692	
Sex (M:F)		2,645:1,635	1,526:1,166	< 0.0001^b^
Age (year)^a^	4,280/2,692	63.0 ± 11.4	50.6 ± 16.2	< 0.0001^b^
BMI (kg/m^2^)^a^	4,280/2,692	24.3 ± 4.1	22.8 ± 3.2	< 0.0001^b^
HbA1c (%)^a^	3,169/571	7.9 ± 2.7	5.3 ± 0.7	< 0.0001^c^
FPG (mmol/L)^a^	1,930/1,139	8.3 ± 2.9	5.3 ± 0.6	< 0.0001^c^
Duration of diabetes (year)^a^	2,891/-	13.7 ± 9.7	-	

^a^ Data are mean ± standard deviation.

^b^ Student’s unpaired t-test

^c^ Mann-Whitney U test

BMI: body mass index, HbA1c: Glycated hemoglobin, FPG: fasting plasma glucose

**Table 2:** Genotype distributions of 10 SNPs in case and control groups.

SNP	Nearby gene^a^	Allele1/Allele2(Risk Allele)	Allele 11/12/22		P for HWE test	
			(%)			
			Type 2 diabetes	Controls	Type 2 diabetes	Controls
rs12571751	*ZMIZ1*	A/G (A)	1233/2098/783	725/1260/580	0.038	0.466
			(30.0/51.0/19.0)	(28.3/49.1/22.6)		
rs10842994	*KLHDC5*	C/T (C)	2878/1193/121	1770/795/93	0.844	0.749
			(68.7/28.5/2.88)	(66.6/29.9/3.5)		
rs2796441	*TLE1*	G/A (G)	620/2032/1532	355/1264/1021	0.201	0.244
			(14.8/48.6/36.6)	(13.4/47.9/38.7)		
rs459193	*ANKRD55*	G/A (G)	971/2108/1065	595/1282/762	0.249	0.208
			(23.4/50.9/25.7)	(22.5/48.6/28.9)		
rs10401969	*CILP2*	C/T (C)	43/753/3411	32/463/2168	0.840	0.198
			(1.0/17.9/81.1)	(1.2/17.4/81.4)		
rs12970134	*MC4R*	A/G (A)	117/1181/2876	66/723/1854	0.748	0.652
			(2.8/28.3/68.9)	(2.5/27.4/70.1)		
rs7202877	*BCAR1*	T/G (T)	2584/1349/193	1645/881/127	0.32	0.517
			(62.6/32.7/4.7)	(62.0/33.2/4.8)		
rs11063069	*CCND2*	A/G (G)	4038/216/3	2552/129/4	0.949	0.080
			(94.86/5.07/0.07)	(95.0/4.8/0.2)		
rs8108269	*GIPR*	G/T (G)	1723/1853/514	1057/1230/333	0.648	0.397
			(42.1/45.3/12.6)	(40.4/46.9/12.7)		
rs8090011	*LAMA1*	C/G (G)	341/1756/2108	213/1149/1308	0.354	0.073
			(8.1/41.8/50.1)	(8.0/43.0/49.0)		

^a^ Information in the original report is shown.

HWE: Hardy-Weinberg equilibrium

Of the 7 SNPs previously shown to be associated with type 2 diabetes in European GWAS un-stratified analyses; rs12571751 in *ZMIZ1*, rs10842994 near *KLHDC5*, rs2796441 near *TLE1*, rs459193 near *ANKRD55*, rs10401969 in *CILP2*, rs12970134 near *MC4R* and rs7202877 near *BCAR1* [16], all 7 SNPs had the same direction of effect (odds ratio > 1.0 adjusted for sex, age and BMI) as in the original reports (p = 7.81 × 10^–3^, binomial test, Table 3, S2 Table). Among them, rs12571751 in *ZMIZ1* was significantly associated with type 2 diabetes even after Bonferroni’s correction in this study (p = 4.1 × 10^–3^, odds ratio [OR] = 1.123, 95% confidence interval [CI] 1.037–1.215, adjusted for sex, age and BMI; Table 3). Rs10842994 near *KLHDC5* was nominally associated with type 2 diabetes, but did not show a statistically significant association after Bonferroni’s correction (0.005 ≤ p < 0.05, adjusted for sex, age and BMI; Table 3). The remaining 5 SNPs were not associated with type 2 diabetes (p ≥ 0.05, adjusted for sex, age and BMI; Table 3). When we constructed a genetic risk score (GRS) by summing the number of risk alleles for the 7 SNPs (GRS-7), the GRS-7 was significantly associated with type 2 diabetes in the present Japanese population (p = 2.3 × 10^–4^, adjusted for sex, age and BMI; Table 3). We also constructed GRS-6 by excluding rs12571751 in *ZMIZ1* from the GRS-7, and observed that the GRS-6 was associated with type 2 diabetes in the Japanese population (p = 7.9 × 10^–3^, adjusted for sex, age and BMI; Table 3).

**Table 3:** Association of 7 SNP loci with type 2 diabetes in a Japanese population.

SNP	Nearby gene^a^	Risk Allele^b^	RAF (case/control)	Unadjusted		Adjusted^c^	
				*p* value	OR(95%CI)	*p* value	OR (95%CI)
rs12571751	*ZMIZ1*	A	0.55/0.53	2.7 × 10^–3^	1.114(1.038–1.195)	4.1 × 10^–3^	1.123 (1.037–1.215)
rs10842994	*KLHDC5*	C	0.83/0.82	0.046	1.096 (1.002–1.199)	0.028	1.120 (1.013–1.238)
rs2796441	*TLE1*	G	0.39/0.37	0.043	1.077 (1.002–1.157)	0.052	1.083 (0.999–1.173)
rs459193	*ANKRD5*	G	0.49/0.47	0.021	1.085 (1.012–1.163)	0.084	1.071 (0.991–1.157)
rs10401969	*CILP2*	C	0.100/0.099	0.884	1.009 (0.900–1.130)	0.719	1.024 (0.900–1.164)
rs12970134	*MC4R*	A	0.170/0.168	0.235	1.058 (0.964–1.161)	0.844	1.011 (0.911–1.121)
rs7202877	*BCAR1*	T	0.790/0.786	0.614	1.022 (0.940–1.111)	0.548	1.029 (0.937–1.130)
GRS-7^d^ ^,^ ^f^				3.0 × 10–^5^	1.071 (1.037–1.106)	2.3 × 10^–4^	1.070 (1.032–1.109)
GRS-6^e^ ^,^ ^f^				1.6 × 10–^3^	1.059 (1.022–1.098)	7.9 × 10^–3^	1.056 (1.014–1.099)

The results of logistic regression analysis are shown.

^a^ Information in the original report is shown.

^b^ Risk allele reported in the previous reports.

^c^ Adjusted for age, sex and BMI.

^d^ The genetic risk score (GRS-7) was calculated according to the number of risk alleles by counting the 7 European genome-wide association study derived SNPs.

^e^ The genetic risk score (GRS-6) was calculated according to the number of risk alleles by counting the 6 European genome-wide association study derived SNPs, rs10842994, rs2796441, rs459193, rs10401969, rs12970134, rs7202877

^f^ Individuals who had complete genotype data for the 7 SNPs were used for the analyses (n = 6,200)

The remaining 3 SNPs, rs11063069 near *CCND2* and rs8108269 near *GIPR*, which were identified in sex-differentiated European GWAS [16], and rs8090011 in *LAMA1*, which was shown to be associated with non-obese European type 2 diabetes [15], were not associated with type 2 diabetes in the un-stratified analyses in this study (p ≥ 0.05, adjusted for sex, age and BMI; Tables 4 and 5).

**Table 4:** Sex differentiated analysis for the association of rs11063069 near *CCND2* and rs8108269 near *GIPR* with type 2 diabetes.

SNP	Nearby gene^a^	Risk Allele^b^	All^c^		Male^d^ (n = 4,171)		Female^d^ (n = 2,801)	
			*P* value	OR(95%CI)	*P* value	OR(95%CI)	*P* value	OR (95%CI)
rs11063069	*CCND2*	G	0.42	1.104(0.868–1.405)	0.962	1.008 (0.741–1.370)	0.291	1.236 (0.834–1.830)
rs8108269	*GIPR*	G	0.369	1.038(0.957–1.125)	0.650	0.976 (0.881–1.083)	0.038	1.152 (1.008–1.318)

^a^ Information in the original report is shown.

^b^ Risk allele reported in the previous reports

^c^ Results of logistic regression analysis with adjustment for age, sex, and BMI are shown

^d^ Results of logistic regression analysis with adjustment for age and BMI are shown

**Table 5:** BMI stratified analysis for the association of rs8090011 in *LAMA1* with type 2 diabetes.

SNP	Nearby gene	Risk Allele^a^	All		BMI < 25		BMI ≥ 25	
					(case = 2,627 control = 2,692)		(case = 1,653 control = 2,692)	
			*P* value	OR(95%CI)	*P* value	OR(95%CI)	*P* value	OR (95%CI)
rs8090011	*LAMA1*	G	0.504	1.030 (0.945–1.122)	0.511	1.034 (0.936–1.141)	0.777	1.020 (0.888–1.173)

Results of logistic regression analysis with adjustment for age, sex and BMI are shown

^a^ Risk allele reported in the previous reports

In subsequent sex-differentiated analyses, rs8108269 near *GIPR* was nominally associated with type 2 diabetes in female, but the association was not significant after Bonferroni’s correction (p = 0.038, adjusted for age and BMI; Table 4, S3 Table). Rs11063069 was not associated with type 2 diabetes in the sex-differentiated analyses (p ≥ 0.05, adjusted for age and BMI; Table 4, S3 Table). In BMI-stratified analyses, we did not observe any association of rs8090011 in *LAMA1* with type 2 diabetes (p ≥ 0.05, adjusted for sex, age and BMI; Table 5, S3 Table). We also performed BMI-stratified analyses using cutoff value of 30 kg/m^2^, but did not observe any association of rs8090011 in *LAMA1* with type 2 diabetes (S4 Table).

We further examined the association of the 10 SNPs with quantitative glycemic traits, HOMA-IR, HOMA-β and FPG using control participants (Tables 6, 7 and 8). Rs11063069-G near *CCND2* was significantly associated with a decrease of HOMA-β in male (p = 0.0048, adjusted for age and BMI; Table 7). Rs8090011-G in *LAMA1* was nominally associated with a decrease of HOMA-β, and with an increase of FPG in un-stratified analyses (0.005 ≤ p < 0.05 adjusted for sex, age and BMI; Table 8). The remaining 8 SNP loci and the GRS-7 were not associated with these glycemic traits in this study (p ≥ 0.05, Tables 6, 7 and 8). We also examined the association of the risk allele of 10 SNP loci with BMI, but none of 10 SNP loci was associated with BMI (p ≥ 0.05 adjusted for sex and age; S5 Table).

**Table 6:** Association of 7 SNP loci with quantitative traits related to glucose metabolism in control individuals.

SNP	Nearby gene^a^	Risk Allele^b^	HOMA-IR^c^ (n = 802)		HOMA-β^c^ (n = 802)		FPG^c^ (n = 1,144)	
			Effect (SE)	*p* value	Effect (SE)	*p* value	Effect (SE)	*p* value
rs12571751	*ZMIZ1*	A	-0.030 (0.024)	0.206	-0.029 (0.025)	0.249	0.002 (0.004)	0.734
rs10842994	*KLHDC5*	C	0.031 (0.030)	0.307	0.026 (0.032)	0.410	0.002 (0.006)	0.756
rs2796441	*TLE1*	G	-0.007 (0.024)	0.773	-0.030 (0.025)	0.241	0.005 (0.004)	0.279
rs459193	*ANKRD5*	G	-0.008 (0.024)	0.719	0.003 (0.024)	0.887	0.003 (0.004)	0.472
rs10401969	*CILP2*	C	0.026 (0.038)	0.501	-0.003 (0.040)	0.945	-0.003 (0.007)	0.715
rs12970134	*MC4R*	A	-0.029 (0.032)	0.362	-0.022 (0.034)	0.515	-0.005 (0.006)	0.356
rs7202877	*BCAR1*	T	-0.017 (0.029)	0.561	-0.036 (0.031)	0.237	4.82×10^–4^ (0.005)	0.928
GRS-7 ^d^			-0.009 (0.011)	0.399	-0.014 (0.011)	0.193	0.001 (0.002)	0.599

The results of linear regression analysis with adjustment for age, sex and BMI are presented.

^a^ Information in the original report is shown.

^b^ The risk allele for type 2 diabetes reported in the previous reports

^c^ Values are log-transformed for the analyses

^d^ The genetic risk score (GRS-7) was calculated according to the number of risk allele of 7 SNPs, and the individuals who had complete genotype data for the 7 SNPs were used for the analysis (n = 716 for HOMA-IR and HOMA-β, n = 1,008 for FPG) adjusted for age, sex and BMI.

**Table 7:** Association of rs11063069 near *CCND2* and rs8108269 near *GIPR* with quantitative traits related to glucose metabolism in male or in female.

SNP	Nearby gene ^a^	Risk allele ^b^	Sex	HOMA-IR ^c^		HOMA-β ^c^		FPG ^c^	
				Effect (SE)	*p* value	Effect (SE)	*P* value	Effect (SE)	*p* value
rs11063069	*CCND2*	G	All ^d^	-0.082 (0.073)	0.265	-0.163(0.077)	0.034	-0.001 (0.014)	0.945
			Male ^e^	-0.133 (0.086)	0.121	-0.256 (0.090)	4.8×10^–3^	0.020 (0.017)	0.26
			Female ^e^	0.037 (0.135)	0.782	0.055 (0.140)	0.694	-0.038 (0.024)	0.11
rs8108269	*GIPR*	G	All ^d^	0.018 (0.024)	0.472	0.023 (0.026)	0.374	0.001 (0.004)	0.805
			Male ^e^	0.024 (0.032)	0.457	0.036 (0.034)	0.281	2.6×10^–4^ (0.006)	0.965
			Female ^e^	0.010 (0.037)	0.779	0.008 (0.039)	0.839	0.002 (0.007)	0.732

^a^ Information in the original report is shown.

^b^ Risk allele reported in the previous reports

^c^ values are log-transformed for the analyses

^d^ Results of logistic regression analysis with adjustment for age, sex, and BMI are shown.

^e^ Results of logistic regression analysis with adjustment for age and BMI are shown.

All; n = 802 for HOMA-IR and HOMA-β, n = 1144 for FPG

Male; n = 480 for HOMA-IR and HOMA-β, n = 651 for FPG

Female; n = 322 for HOMA-IR and HOMA-β, n = 493 for FPG

**Table 8:** Association of rs8090011 in *LAMA1* with quantitative traits related to glucose metabolism in obese controls (BMI ≥ 25) or in non-obese controls (BMI < 25).

SNP	Nearby gene	Risk allele^a^	BMI	HOMA-IR^b^		HOMA-β^b^		FPG^b^	
				Effect (SE)	*p* value	Effect (SE)	*p* value	Effect (SE)	*p* value
rs8090011	*LAMA1*	G	All	-0.029 (0.027)	0.284	-0.058 (0.028)	0.04	0.010 (0.005)	0.049
			BMI < 25	-0.003 (0.037)	0.945	-0.030 (0.039)	0.449	0.011 (0.008)	0.159
			BMI ≥ 25	-0.066 (0.039)	0.09	-0.076 (0.039)	0.054	0.006 (0.006)	0.286

Results of linear regression analysis with adjustment for age, sex and BMI are presented.

^a^ Risk allele reported in the previous reports

^b^ values are log-transformed for the analysis

BMI < 25; n = 428 for HOMA-IR and HOMA-β, n = 482 for FPG

BMI ≥ 25; n = 374 for HOMA-IR and HOMA-β, n = 662 for FPG

## Discussion

In this study, we examined the association of 7 SNPs, rs12571751 in *ZMIZ1*, rs10842994 near *KLHDC5*, rs2796441 near *TLE1*, rs459193 near *ANKRD55*, rs10401969 in *CILP2*, rs12970134 near *MC4R*, and rs7202877 near *BCAR1*, with susceptibility to type 2 diabetes in a Japanese population, and found that rs12571751 in *ZMIZ1* was significantly associated with type 2 diabetes in the Japanese population. We also examined the effects of 3 additional SNP loci, rs11063069 near *CCND2* and rs8108269 near *GIPR*, which were identified in sex-differentiated analyses [16], and rs8090011 in *LAMA1*, which was shown to be associated with non-obese type 2 diabetes in European GWAS [15], but we did not replicate the original association in the Japanese population (Tables 4 and 5, S3 Table).

Genome-wide association studies for type 2 diabetes have been conducted extensively in different ethnic groups, and have successfully identified over 80 susceptibility loci thus far [1–19]. Among them, over 50 loci were identified by GWAS in European populations [1–6,9,10,15, 16], and many of the European GWAS-derived loci have been shown to be associated with type 2 diabetes in other ethnic groups, whereas others did not have significant effects in populations of non-European origin [8,11,18,20–29]. In some cases, the sample size was not sufficient, but there may be significant interethnic differences in the effect sizes at several loci; therefore, it is important to evaluate the effect of each locus in different ethnic groups [18,27].

Among 7 SNPs identified in un-stratified European GWAS meta-analyses, rs12571751 in *ZMIZ1* was significantly associated with type 2 diabetes in the present Japanese population, indicating that this locus was common susceptibility locus for type 2 diabetes across different ethnic groups. The effect size for rs12571751 in this study (OR = 1.12) was comparable with those for the previously reported type 2 diabetes loci in Japanese populations [36], but not as large as that of *TCF7L2* (OR = 1.51) or *KCNQ1* (OR = 1.42) (S6 Table). Rs12571751 is located at intron 5 of *ZMIZ1*. *ZMIZ1* has been shown to express ubiquitously in human tissues, including pancreas [37]. *ZMIZ1* encodes a member of the PIAS (protein inhibitor of activated STAT) family proteins, which interact with p53 [38], and a recent report using β-cell specific p53 deleted mice showed that p53 depletion could prevent glucotoxic β-cell death in these mice [39]; therefore, *ZMIZ1* might be involved in β-cell death through p53 activation and contribute to conferring susceptibility to type 2 diabetes.

The directions of the effects for the remaining 6 SNPs were consistent with those in the original reports [16] (p = 1.56 × 10^–2^, binomial test; Table 3), and the GRS-6 constructed from the 6 SNPs was associated with type 2 diabetes (p = 7.9 × 10^–3^, adjusted for sex, age and BMI; Table 3).

Since the association of GRS-7, which included the information of these 6 SNPs in addition to that of rs12571751 in *ZMIZ1* locus, with the disease was stronger than that of rs12571751 alone, it is suggested that these 6 loci have some effects on conferring susceptibility to type 2 diabetes also in the Japanese population.

With regard to rs8108269 near *GIPR* and rs11063069 near *CCND2*, the association of the *GIPR* locus with type 2 diabetes was more significant in female in the original European study, whereas the effect of *CCND2* locus was stronger in male [16]. Rs8108269 near *GIPR* was nominally associated with type 2 diabetes in female, suggesting that *GIPR* locus might be involved in the developement of type 2 diabetes in female in the Japanese population similar as that observed in the European population. Because the risk allele frequency of rs11063069 near *CCND2* was lower in the Japanese population (RAF = 1.0%) than in European populations (RAF = 20.7%), and estimated power for the present study to replicate the original finding in European GWAS was insufficient for this SNP (13% for rs11063069; S7 Table). Furthermore, the risk allele of rs11063069 was significantly associated with a decrease of HOMA-β in male (p = 0.0048 adjusted for age and BMI; Table 7); therfore, the risk allele of *CCND2* might have some effects on the decrease of insulin secretion, and on conferring susceptibility to type 2 diabetes in male, as reported in the original European GWAS.

The *LAMA1* locus has been shown to be associated with lean type 2 diabetes, but not with obese type 2 diabetes, in the European GWAS [15]. We did not replicate the original associations (p ≥ 0.05 adjusted for sex, age and BMI; Table 5, S3 Table). Because the present study had sufficient power to replicate the original findings for the *LAMA1* locus (90%; S7 Table), we considered that the effect of rs8090011-G in *LAMA1* locus was not major in the Japanese population. These 3 SNP loci need to be evaluated further in larger Japanese cohorts.

Results found in the case-control sample were not always consistent with the findings from quantitative trait analyses in this study, a sample size of quantitative traits analyses in this study was not sufficiently large enough, but it has been shown that genetic loci for glycemic traits found in non-diabetic controls were not always linked to type 2 diabetes risk [9, 40]; therefore, the biological significance of each locus need to be evaluated by alternative approaches.

In the present study, we did not observe any association with type 2 diabetes except for *ZMIZ1* and *KLHDC5* loci in a Japanese population (p ≥ 0.05 adjusted for sex, age and BMI; Table 3). Because the genotyping success rates for all of the SNPs were over 95.8%, and the genotype concordance rates were 100% (see Materials and Methods, S1 Table), the discrepancy was not considered to result from technical issues.

We also evaluated heterogeneity in terms of genotype distributions for each SNP among individual collections bacause we recruited case and control individuals from 6 and 4 distinct institutions in this study, respectively. The results indicated that there is no heterogeneity in genotype distributions among each case or control collection (S8 Table).

In this study, the control individuals were significantly younger than the type 2 diabetes patients (Table 1); therefore our control group may have included individuals who will develop the disease later, which may increase the possibility of a type 2 error, although we included age as a co-variable in the logistic regression model. Therefore, we evaluated the association of the 10 SNPs with type 2 diabetes using older control individuals (age ≥ 40 years, ≥ 50 years, or ≥ 60 years). The effect sizes of most SNP loci or the GRS-7 examined in this study increased when we used older control individuals for the association study (S9 Table). Because the effect directions of most SNPs were consistent with our original findings, we consider that our conclusions were not skewed by the inclusion of these younger control individuals, although inclusion of patients who will develop the disease later may have reduced the statistical power of our study. Because the estimated power in the present study for the non-replicated SNPs did not reach 80%, except for rs8090011 in *LAMA1* (S7 Table), insufficient sample size may be a principal explanation for the discrepancies between the present study and the original European studies.

In summary, we examined the association of 10 SNPs, previously identified in European GWAS, with type 2 diabetes in an independent Japanese population. Our results suggested that rs12571751 in *ZMIZ1* has a significant effect on conferring susceptibility to type 2 diabetes also in the Japanese. The remaining 9 SNP loci did not show significant effects in the Japanese population by themselves, although the effects of these SNP loci need to be evaluated in sufficiently powered, larger Japanese populations.

## Supporting Information

S1 Table Information of genotyping success rates for individual 10 SNPs. ^a^ Information in the original report is shown. ^b^ Risk allele reported in the previous reports.
(DOCX)

S2 Table Association of 7 SNP loci with type 2 diabetes in a Japanese population and original reports. The results of logistic regression analysis are shown. ^a^ Information in the original report is shown. ^b^ Risk allele reported in the previous reports. ^c^ Adjusted for age, sex and BMI. ^d^ Information in the original European GWAS (Morris AP et al. *Nat Genet* 44: 981–990, 2012) is shown. ^e^ The genetic risk score (GRS-7) was calculated according to the number of risk alleles by counting the 7 European genome-wide association study derived SNPs. ^f^ The genetic risk score (GRS-6) was calculated according to the number of risk alleles by counting the 6 European genome-wide association study derived SNPs, rs10842994, rs2796441, rs459193, rs10401969, rs12970134, rs7202877. ^g^ Individuals who had complete genotype data for the 7 SNPs were used for the analyses (n = 6,200).
(DOCX)

S3 Table Association of 3 SNP loci with type 2 diabetes in a Japanese population and original reports. Results of logistic regression analysis with adjustment for age and BMI (rs11063069 and rs8108269) or age, sex and BMI (rs8090011) are shown. ^a^ Information in the original European GWAS (Morris AP et al. *Nat Genet* 44: 981–990, 2012, Perry JR et al *PLoS Genet* 8:e1002741, 2012). ^b^ Information in the present Japanese analyses. ^c^ Risk allele frequency in un-stratified group. ^d^ Association data in each stratified group.
(DOCX)

S4 Table BMI stratified analysis for the association of *LAMA1*with type 2 diabetes. Results of logistic regression analysis with adjustment for age, sex and BMI are shown. ^a^ Risk allele reported in the previous reports. ^b^ Results of logistic regression analysis with adjustment for age, sex and BMI are shown.
(DOCX)

S5 Table Association of 10 SNP loci with BMI in control individuals. The results of linear regression analysis with adjustment for age and sex are presented. ^a^ Information in the original report is shown. ^b^ The risk allele for type 2 diabetes reported in the previous reports. ^c^ Values are log-transformed for the analyses.
(DOCX)

S6 Table Effect sizes for the 10 SNPs in this study and other known type 2 diabetes-related SNPs in Japanese populations. The 10 SNPs examined in the present study are shown in bold. *Effect sizes for known type 2 diabetes-related SNPs, which were previously shown to be associated with type 2 diabetes (p < 0.05) in Japanese populations. Data from a previous report (Imamura M et al. *J Clin Endocrinol Metab*. 2013 98(10)) is shown.
(DOCX)

S7 Table Power estimation for each SNP locus to replicate the results of original European study in the present study. Power estimation was performed using CaTS power calculator, CaTS: http://www.sph.umich.edu/csg/abecasis/CaTS/). The prevalence of type 2 diabetes is assumed to be 10%, α = 0.05. ^a^ Information in the original report is shown. ^b^ Risk allele for type 2 diabetes reported in the previous report. ^c^ Risk allele frequency in the Japanese population (controls) in the present study.
(DOCX)

S8 Table Comparison of risk allele frequencies among individual areas for sample collection. ^a^ collection 1: BioBank Japan; collection 2: Juntendo University; collection 3: Kawasaki Medical School; collection 4: case Shiga University of Medical Science, control Keio University; collection 5: St. Marianna University; collection 6: Toyama University; collection 7: Japan SNP data base. ^b^ Chi square test.
(DOCX)

S9 Table Association study of 10 SNPs with type 2 diabetes using older control (age ≥ 40, n = 1,928, age ≥ 50, n = 1,489, age ≥ 60 n = 845). Results of logistic regression analysis using all type 2 diabetes participants (n = 4,280) are shown. ^a^ Information in the original report is shown. ^b^ Risk allele reported in the previous reports. ^c^ Adjusted for age, sex and BMI. ^d^ GRS-7 was calculated according to the number of risk allele of the 7 SNPs, and the individuals having complete genotype data for the 7 SNPs were used for the analysis (age ≥ 40, n = 5,498, age ≥ 50, n = 5,118, age ≥ 60 n = 4,545) adjusted for age, sex and BMI.
(DOCX)
