# Supplementary material for: Replication Study in a Japanese Population to Evaluate the Association between 10 SNP Loci, Identified in European Genome-Wide Association Studies, and Type 2 Diabetes
Source: PLoS One. 2015 May 7;10(5):e0126363. doi: 10.1371/journal.pone.0126363 (PMC4423838; doi:10.1371/journal.pone.0126363)
Supplement: S1 Table — a Information in the original report is shown. b Risk allele reported in the previous reports. (DOCX) [file pone.0126363.s001.docx]

**Table S1.** Information of genotyping success rates for individual 10 SNPs

| SNP | Nearby Gene^a^ | Risk alleles^b^ | Success rate |
| --- | --- | --- | --- |
| rs12571751 | *ZMIZ1* | A | 95.8 % |
| rs10842994 | *KLHDC5* | C | 98.3 % |
| rs2796441 | *TLE1* | G | 97.9 % |
| rs459193 | *ANKRD55* | G | 97.3 % |
| rs10401969 | *CILP2* | C | 98.5 % |
| rs12970134 | *MC4R* | A | 97.8 % |
| rs7202877 | *BCAR1* | T | 98.1 % |
| rs11063069 | *CCND2* | G | 99.6 % |
| rs8108269 | *GIPR* | G | 97.1 % |
| rs8090011 | *LAMA1* | G | 98.6 % |

^a^ Information in the original report is shown

^b^ Risk allele reported in the previous reports
