# Supplementary material for: Replication Study in a Japanese Population to Evaluate the Association between 10 SNP Loci, Identified in European Genome-Wide Association Studies, and Type 2 Diabetes
Source: PLoS One. 2015 May 7;10(5):e0126363. doi: 10.1371/journal.pone.0126363 (PMC4423838; doi:10.1371/journal.pone.0126363)
Supplement: S2 Table — The results of logistic regression analysis are shown. a Information in the original report is shown. b Risk allele reported in the previous reports. c Adjusted for age, sex and BMI. d Information in the original European GWAS (Morris AP et al. Nat Genet 44: 981–990, 2012) is shown. e The genetic risk score (GRS-7) was calculated according to the number of risk alleles by counting the 7 European genome-wide association study derived SNPs. f The genetic risk score (GRS-6) was calculated according to the number of risk alleles by counting the 6 European genome-wide association study derived SNPs, rs10842994, rs2796441, rs459193, rs10401969, rs12970134, rs7202877. g Individuals who had complete genotype data for the 7 SNPs were used for the analyses (n = 6,200). (DOCX) [file pone.0126363.s002.docx]

**Table S2.** Association of 7 SNP loci with type 2 diabetes in a Japanese population and original reports

| SNP | Nearby  gene^a^ | Risk  Allele^b^ | RAF  (case/control) | Present study^c^ | | RAF^d^  (original) | Original reports^d^ | |
| --- | --- | --- | --- | --- | --- | --- | --- | --- |
|  |  |  |  | *p* value | OR(95%CI) |  | *p* value | OR(95%CI) |
| rs12571751 | *ZMIZ1* | A | 0.55/0.53 | 4.1×10^-3^ | 1.123 (1.037–1.215) | 0.52 | 1.0×10^-10^ | 1.08 (1.05–1.10) |
| rs10842994 | *KLHDC5* | C | 0.83/0.82 | 0.028 | 1.120 (1.013–1.238) | 0.80 | 6.1×10^-10^ | 1.10 (1.06–1.13) |
| rs2796441 | *TLE1* | G | 0.39/0.37 | 0.052 | 1.083 (0.999–1.173) | 0.57 | 5.4×10^-9^ | 1.07 (1.05–1.10) |
| rs459193 | *ANKRD5* | G | 0.49/0.47 | 0.084 | 1.071 (0.991–1.157) | 0.70 | 6.0×10^-9^ | 1.08 (1.05–1.11) |
| rs10401969 | *CILP2* | C | 0.100/0.099 | 0.719 | 1.024 (0.900–1.164) | 0.08 | 7.0×10^-9^ | 1.13 (1.09–1.18) |
| rs12970134 | *MC4R* | A | 0.170/0.162 | 0.844 | 1.011 (0.911–1.121) | 0.27 | 1.2×10^-8^ | 1.08 (1.05–1.10) |
| rs7202877 | *BCAR1* | T | 0.790/0.786 | 0.548 | 1.029 (0.937–1.130) | 0.89 | 3.5×10^-8^ | 1.12 (1.07–1.16) |
| GRS-7^e.g^ |  |  |  | 2.3×10^-4^ | 1.070 (1.032–1.109) |  | - | - |
| GRS-6^f.g^ |  |  |  | 7.9×10^-3^ | 1.056 (1.014-1.099) |  | - | - |

The results of logistic regression analysis are shown

^a^ Information in the original report is shown

^b^ Risk allele reported in the previous reports

^c^ Adjusted for age, sex and BMI

^d^ Information in the original European GWAS (Morris AP et al. *Nat Genet* 44: 981–990, 2012) is shown

^e^ The genetic risk score (GRS-7) was calculated according to the number of risk alleles by counting the 7 European genome-wide association study derived SNPs

^f^ The genetic risk score (GRS-6) was calculated according to the number of risk alleles by counting the 6 European genome-wide association study derived SNPs, rs10842994, rs2796441, rs459193, rs10401969, rs12970134, rs7202877

^g^ Individuals who had complete genotype data for the 7 SNPs were used for the analyses (n = 6,200)
