# Supplementary material for: Replication Study in a Japanese Population to Evaluate the Association between 10 SNP Loci, Identified in European Genome-Wide Association Studies, and Type 2 Diabetes
Source: PLoS One. 2015 May 7;10(5):e0126363. doi: 10.1371/journal.pone.0126363 (PMC4423838; doi:10.1371/journal.pone.0126363)
Supplement: S3 Table — Results of logistic regression analysis with adjustment for age and BMI (rs11063069 and rs8108269) or age, sex and BMI (rs8090011) are shown. a Information in the original European GWAS (Morris AP et al. Nat Genet 44: 981–990, 2012, Perry JR et al PLoS Genet 8:e1002741, 2012). b Information in the present Japanese analyses. c Risk allele frequency in un-stratified group. d Association data in each stratified group. (DOCX) [file pone.0126363.s003.docx]

**Table S3**. Association of 3 SNP loci with type 2 diabetes in a Japanese population and original reports

| SNP | Nearby gene ^a^ | Risk Allele ^a^ | RAF ^b, c^  (case  /control) | RAF ^a, c^  (Original) | Stratified group | Present study ^b, d^ | | Original reports ^a, d^ | |
| --- | --- | --- | --- | --- | --- | --- | --- | --- | --- |
|  |  |  |  |  |  | *p* value | OR(95%CI) | *p* value | OR(95%CI) |
| rs11063069 | *CCND2* | G | 0.0260  /0.0255 | 0.21 | male | 0.962 | 1.008 (0.741-1.370) | 1.1×10^-9^ | 1.12  (1.08-1.16) |
| rs8108269 | *GIPR* | G | 0.65  /0.64 | 0.31 | female | 0.038 | 1.152 (1.008-1.318) | 2.2×10^-7^ | 1.10  (1.06-1.14) |
| rs8090011 | *LAMA1* | G | 0.710  /0.705 | 0.38 | BMI < 25 | 0.511 | 1.034 (0.936-1.141) | 8.4×10^-9^ | 1.13  (1.09-1.18) |

Results of logistic regression analysis with adjustment for age and BMI (rs11063069 and rs8108269) or age, sex and BMI (rs8090011) are shown

^a^ Information in the original European GWAS (Morris AP et al. *Nat Genet* 44: 981–990, 2012, Perry JR et al *PLoS Genet* 8:e1002741, 2012)

^b^ Information in the present Japanese analyses

^c^ Risk allele frequency in un-stratified group

^d^ Association data in each stratified group
