# Supplementary material for: Replication Study in a Japanese Population to Evaluate the Association between 10 SNP Loci, Identified in European Genome-Wide Association Studies, and Type 2 Diabetes
Source: PLoS One. 2015 May 7;10(5):e0126363. doi: 10.1371/journal.pone.0126363 (PMC4423838; doi:10.1371/journal.pone.0126363)
Supplement: S4 Table — Results of logistic regression analysis with adjustment for age, sex and BMI are shown. a Risk allele reported in the previous reports. b Results of logistic regression analysis with adjustment for age, sex and BMI are shown. (DOCX) [file pone.0126363.s004.docx]

**Table S4.** BMI stratified analysis for the association of *LAMA1*with type 2 diabetes

| SNP | Risk ^a^ | All^b^ | | BMI < 30^b^  (case= 3,918  control= 2,692) | | BMI ≥ 30^b^  (case= 362  control= 2,692) | |
| --- | --- | --- | --- | --- | --- | --- | --- |
|  |  | *P* value | OR  (95%CI) | *P* value | OR  (95%CI) | *P* value | OR  (95%CI) |
| rs8090011 | G | 0.504 | 1.030  (0.945-1.122) | 0.555 | 1.027  (0.941-1.120) | 0.457 | 1.156  (0.789-1.693) |

Results of logistic regression analysis with adjustment for age, sex and BMI are shown

^a^ Risk allele reported in the previous reports

^b^ Results of logistic regression analysis with adjustment for age, sex and BMI are shown
