# Supplementary material for: Replication Study in a Japanese Population to Evaluate the Association between 10 SNP Loci, Identified in European Genome-Wide Association Studies, and Type 2 Diabetes
Source: PLoS One. 2015 May 7;10(5):e0126363. doi: 10.1371/journal.pone.0126363 (PMC4423838; doi:10.1371/journal.pone.0126363)
Supplement: S5 Table — The results of linear regression analysis with adjustment for age and sex are presented. a Information in the original report is shown. b The risk allele for type 2 diabetes reported in the previous reports. c Values are log-transformed for the analyses. (DOCX) [file pone.0126363.s005.docx]

**Table S5.** Association of 10 SNP loci with BMI in control individuals

| SNP | Nearby gene ^a^ | Risk Allele ^b^ |  | BMI^c^ (n=1228) | |
| --- | --- | --- | --- | --- | --- |
|  |  |  |  | Effect (SE) | *p* value |
| rs12571751 | *ZMIZ1* | A |  | 0.008 (0.006) | 0.148 |
| rs10842994 | *KLHDC5* | C |  | 0.002 (0.007) | 0.786 |
| rs2796441 | *TLE1* | G |  | 0.007 (0.006) | 0.232 |
| rs459193 | *ANKRD5* | G |  | 0.005 (0.005) | 0.377 |
| rs10401969 | *CILP2* | C |  | -0.011 (0.009) | 0.214 |
| rs12970134 | *MC4R* | A |  | -0.007 (0.008) | 0.364 |
| rs7202877 | *BCAR1* | T |  | -0.008 (0.007) | 0.226 |
| rs11063069 | *CCND2* | G | All | -0.002 (0.018) | 0.894 |
|  |  |  | male | 0.008(0.021) | 0.699 |
|  |  |  | female | -0.015(0.03) | 0.602 |
| rs8108269 | *GIPR* | G | All | -0.006 (0.006) | 0.258 |
|  |  |  | male | -0.003(0.007) | 0.368 |
|  |  |  | female | -0.011(0.009) | 0.237 |
| rs8090011 | *LAMA1* | G | All | -0.011 (0.006) | 0.073 |
|  |  |  | BMI < 25 | -0.003(0.005) | 0.628 |
|  |  |  | BMI ≥ 25 | -0.007(0.007) | 0.347 |
|  |  |  | BMI < 30 | -0.007(0.006) | 0.221 |
|  |  |  | BMI ≥ 30 | -0.004(0.013) | 0.745 |

The results of linear regression analysis with adjustment for age and sex are presented

^a^ Information in the original report is shown

^b^ The risk allele for type 2 diabetes reported in the previous reports

^c^ Values are log-transformed for the analyses
