# Supplementary material for: Replication Study in a Japanese Population to Evaluate the Association between 10 SNP Loci, Identified in European Genome-Wide Association Studies, and Type 2 Diabetes
Source: PLoS One. 2015 May 7;10(5):e0126363. doi: 10.1371/journal.pone.0126363 (PMC4423838; doi:10.1371/journal.pone.0126363)
Supplement: S6 Table — The 10 SNPs examined in the present study are shown in bold. *Effect sizes for known type 2 diabetes-related SNPs, which were previously shown to be associated with type 2 diabetes (p < 0.05) in Japanese populations. Data from a previous report (Imamura M et al. J Clin Endocrinol Metab. 2013 98(10)) is shown. (DOCX) [file pone.0126363.s006.docx]

**Table S6.** Effect sizes for the 10 SNPs in this study and other known type 2 diabetes-related SNPs in Japanese populations

| **Locus** | **Marker** | **odds ratio(95%CI)** |  |
| --- | --- | --- | --- |
|  |  | **Japanese** |  |
| *TCF7L2* | rs12255372 | 1.51(1.21-1.89) | * |
| *KCNQ1* | rs2237897 | 1.42(1.31-1.53) | * |
| *DUSP9* | rs5945326 | 1.37(1.22-1.54) | * |
| *CDKN2A/2B* | rs10811661 | 1.35(1.25-1.45) | * |
| *CDKAL1* | rs7754840 | 1.29(1.20-1.39) | * |
| *IGF2BP2* | rs1470579 | 1.28(1.18-1.38) | * |
| *IRS1* | rs2943641 | 1.22(1.07-1.39) | * |
| *SLC30A8* | rs13266634 | 1.21(1.12-1.30) | * |
| *HHEX* | rs1111875 | 1.19(1.10-1.29) | * |
| *UBE2E2* | rs9812056 | 1.19(1.08-1.31) | * |
| *ANK1* | rs515071 | 1.18(1.08-1.29) | * |
| *JAZF1* | rs864745 | 1.18(1.08-1.29) | * |
| *MAEA* | rs7656416 | 1.17(1.09-1.26) | * |
| *CDC123/CAMK1D* | rs10906115 | 1.17(1.09-1.25) | * |
| *FTO* | rs8050136 | 1.17(1.07-1.28) | * |
| *ZFAND6* | rs11634397 | 1.17(1.04-1.31) | * |
| *HNF1B* | rs7501939 | 1.16(1.07-1.25) | * |
| ***GIPR*** | **rs8108269** | **1.15(1.008-1.318)** |  |
| *GCKR* | rs780094 | 1.14(1.05-1.23) | * |
| ***LAMA1*** | **rs8090011** | **1.13(1.09-1.18)** |  |
| *KCNQ1* | rs231362 | 1.13(1.005-1.29) | * |
| ***ZMIZ1*** | **rs12571751** | **1.12(1.04-1.21)** |  |
| *C2CD4A/B* | rs7172432 | 1.12(1.04-1.20) | * |
| *SPRY2* | rs1359790 | 1.12(1.03-1.21) | * |
| *KCNJ11* | rs5219 | 1.12(1.03-1.20) | * |
| ***KLHDC5*** | **rs10842994** | **1.12(1.01-1.24)** |  |
| *PSMD6* | rs831571 | 1.10(1.03-1.18) | * |
| *DGKB/TMEM195* | rs2191349 | 1.10(1.02-1.20) | * |
| *KLF14* | rs972283 | 1.10(1.02-1.20) | * |
| *ZFAND3* | rs9470794 | 1.10(1.01-1.20) | * |
| *GRB14* | rs3923113 | 1.10(0.99-1.23) | * |
| *PEPD* | rs3786897 | 1.09(1.02-1.17) | * |
| *HMG20A* | rs7178572 | 1.08(1.01-1.16) | * |
| ***TLE1*** | **rs2796441** | **1.08(1.00-1.17)** |  |
| *FITM2-R3HDML-HNF4A* | rs6017317 | 1.07(1.003-1.15) | * |
| ***ANKRD55*** | **rs459193** | **1.07(0.99-1.16)** |  |
| ***BCAR1*** | **rs7202877** | **1.03(0.94-1.13)** |  |
| ***CILP2*** | **rs10401969** | **1.02(0.90-1.16)** |  |
| ***MC4R*** | **rs12970134** | **1.01(0.91-1.12)** |  |
| ***CCND2*** | **rs11063069** | **1.008(0.741-1.37)** |  |

The 10 SNPs examined in the present study are shown in bold

*Effect sizes for known type 2 diabetes-related SNPs, which were previously shown to be associated with type 2 diabetes (p < 0.05) in Japanese populations．Data from a previous report (Imamura M et al. *J Clin Endocrinol Metab*. 2013 98(10)) is shown．
