# Supplementary material for: Replication Study in a Japanese Population to Evaluate the Association between 10 SNP Loci, Identified in European Genome-Wide Association Studies, and Type 2 Diabetes
Source: PLoS One. 2015 May 7;10(5):e0126363. doi: 10.1371/journal.pone.0126363 (PMC4423838; doi:10.1371/journal.pone.0126363)
Supplement: S7 Table — Power estimation was performed using CaTS power calculator, CaTS: http://www.sph.umich.edu/csg/abecasis/CaTS/). The prevalence of type 2 diabetes is assumed to be 10%, α = 0.05. a Information in the original report is shown. b Risk allele for type 2 diabetes reported in the previous report. c Risk allele frequency in the Japanese population (controls) in the present study. (DOCX) [file pone.0126363.s007.docx]

**Table S7.** Power estimation for each SNP locus to replicate the results of original European study in the present study

| SNP | Nearby Gene^a^ | Risk alleles^b^ | RAF ^c^ | Reported OR | Power |
| --- | --- | --- | --- | --- | --- |
| rs12571751 | *ZMIZ1* | A | 0.528 | 1.08(1.05-1.10) | 65% |
| rs10842994 | *KLHDC5* | C | 0.816 | 1.10(1.06-1.13) | 58% |
| rs2796441 | *TLE1* | G | 0.374 | 1.07(1.05-1.10) | 54% |
| rs459193 | *ANKRD55* | G | 0.468 | 1.08(1.05-1.11) | 66% |
| rs10401969 | *CILP2* | C | 0.099 | 1.13(1.09-1.18) | 66% |
| rs12970134 | *MC4R* | A | 0.162 | 1.08(1.05-1.11) | 44% |
| rs7202877 | *BCAR1* | T | 0.786 | 1.12(1.07-1.16) | 76% |
| rs11063069 | *CCND2* | G | 0.026 | 1.08(1.05-1.11) | 13% |
| rs8108269 | *GIPR* | G | 0.638 | 1.07(1.04-1.10) | 51% |
| rs8090011 | *LAMA1* | G | 0.705 | 1.13(1.09-1.18) | 90% |

Power estimation was performed using CaTS power calculator, CaTS: http://www.sph.umich.edu/csg/abecasis/CaTS/)

The prevalence of type 2 diabetes is assumed to be 10%, α = 0.05

^a^ Information in the original report is shown

^b^ Risk allele for type 2 diabetes reported in the previous report

^c^ Risk allele frequency in the Japanese population (controls) in the present study
