# Supplementary material for: Replication Study in a Japanese Population to Evaluate the Association between 10 SNP Loci, Identified in European Genome-Wide Association Studies, and Type 2 Diabetes
Source: PLoS One. 2015 May 7;10(5):e0126363. doi: 10.1371/journal.pone.0126363 (PMC4423838; doi:10.1371/journal.pone.0126363)
Supplement: S8 Table — a collection 1: BioBank Japan; collection 2: Juntendo University; collection 3: Kawasaki Medical School; collection 4: case Shiga University of Medical Science, control Keio University; collection 5: St. Marianna University; collection 6: Toyama University; collection 7: Japan SNP data base. b Chi square test. (DOCX) [file pone.0126363.s008.docx]

**Table S8.** Comparison of risk allele frequencies among individual areas for sample collection

| SNP |  | Risk allele frequencies | | | | | | | *P* ^b^ |
| --- | --- | --- | --- | --- | --- | --- | --- | --- | --- |
|  | collection ^a^ | 1 | 2 | 3 | 4 | 5 | 6 | 7 |  |
| rs12571751 | case | 0.558 | 0.554 | 0.593 | 0.525 | 0.570 | 0.548 |  | 0.391 |
|  | control |  |  |  | 0.530 | 0.529 | 0.513 | 0.539 | 0.798 |
| rs10842994 | case | 0.819 | 0.835 | 0.838 | 0.845 | 0.833 | 0.818 |  | 0.680 |
|  | control |  |  |  | 0.831 | 0.822 | 0.793 | 0.818 | 0.359 |
| rs2796441 | case | 0.402 | 0.387 | 0.389 | 0.383 | 0.396 | 0.382 |  | 0.914 |
|  | control |  |  |  | 0.373 | 0.38 | 0.377 | 0.368 | 0.966 |
| rs459193 | case | 0.492 | 0.507 | 0.48 | 0.481 | 0.499 | 0.470 |  | 0.778 |
|  | control |  |  |  | 0.455 | 0.469 | 0.467 | 0.478 | 0.873 |
| rs10401969 | case | 0.096 | 0.097 | 0.12 | 0.102 | 0.097 | 0.097 |  | 0.817 |
|  | control |  |  |  | 0.095 | 0.089 | 0.103 | 0.105 | 0.758 |
| rs12970134 | case | 0.158 | 0.174 | 0.195 | 0.176 | 0.142 | 0.181 |  | 0.278 |
|  | control |  |  |  | 0.143 | 0.152 | 0.17 | 0.173 | 0.436 |
| rs7202877 | case | 0.787 | 0.789 | 0.80 | 0.783 | 0.796 | 0.792 |  | 0.987 |
|  | control |  |  |  | 0.793 | 0.777 | 0.794 | 0.782 | 0.858 |
| rs8108269 | case | 0.648 | 0.641 | 0.64 | 0.636 | 0.658 | 0.665 |  | 0.884 |
|  | control |  |  |  | 0.637 | 0.634 | 0.656 | 0.628 | 0.726 |
| rs8090011 | case | 0.712 | 0.718 | 0.712 | 0.700 | 0.710 | 0.708 |  | 0.989 |
|  | control |  |  |  | 0.693 | 0.715 | 0.724 | 0.693 | 0.494 |
| rs11063069 | case | 0.032 | 0.024 | 0.024 | 0.023 | 0.028 | 0.020 |  | 0.562 |
|  | control |  |  |  | 0.020 | 0.033 | 0.026 | 0.023 | 0.499 |

^a^ collection 1: BioBank Japan; collection 2: Juntendo University; collection 3: Kawasaki Medical School; collection 4: case Shiga University of Medical Science, control Keio University; collection 5 : St. Marianna University ; collection 6: Toyama University; collection 7: Japan SNP data base

^b^ Chi square test
