# Supplementary material for: Replication Study in a Japanese Population to Evaluate the Association between 10 SNP Loci, Identified in European Genome-Wide Association Studies, and Type 2 Diabetes
Source: PLoS One. 2015 May 7;10(5):e0126363. doi: 10.1371/journal.pone.0126363 (PMC4423838; doi:10.1371/journal.pone.0126363)
Supplement: S9 Table — Results of logistic regression analysis using all type 2 diabetes participants (n = 4,280) are shown. a Information in the original report is shown. b Risk allele reported in the previous reports. c Adjusted for age, sex and BMI. d GRS-7 was calculated according to the number of risk allele of the 7 SNPs, and the individuals having complete genotype data for the 7 SNPs were used for the analysis (age ≥ 40, n = 5,498, age ≥ 50, n = 5,118, age ≥ 60 n = 4,545) adjusted for age, sex and BMI. (DOCX) [file pone.0126363.s009.docx]

**Table S9.** Association study of 10 SNPs with type 2 diabetes using older control (age ≥ 40, n = 1,928, age ≥ 50, n = 1,489, age ≥ 60 n = 845)

| SNP | Nearby  Gene ^a^ | Risk  Allele ^b^ | Control | Unadjusted | | Adjusted ^c^ | |
| --- | --- | --- | --- | --- | --- | --- | --- |
|  |  |  |  | *p* value | OR (95%CI) | *p* value | OR (95%CI) |
| rs12571751 | *ZMIZ1* | A | age ≥ 40 | 0.019 | 1.099 (1.016–1.189) | 0.012 | 1.110(1.024-1.204) |
|  |  |  | age ≥ 50 | 2.8×10^-3^ | 1.141 (1.046–1.244) | 2.3×10^-3^ | 1.145(1.050-1.249) |
|  |  |  | age ≥ 60 | 3.7×10^-5^ | 1.254 (1.126-1.397) | 1.5×10^-5^ | 1.277(1.143-1.426) |
| rs10842994 | *KLHDC5* | C | age ≥ 40 | 0.048 | 1.106 (1.001–1.221) | 0.038 | 1.115(1.006-1.235) |
|  |  |  | age ≥ 50 | 0.053 | 1.113 (0.999–1.241) | 0.051 | 1.116(1.000-1.245) |
|  |  |  | age ≥ 60 | 0.016 | 1.179 (1.031-1.348) | 0.022 | 1.175(1.024-1.349) |
| rs2796441 | *TLE1* | G | age ≥ 40 | 0.073 | 1.076 (0.993–1.165) | 0.053 | 1.085(0.999-1.178) |
|  |  |  | age ≥ 50 | 0.086 | 1.080 (0.989–1.178) | 0.049 | 1.093(1.000-1.194) |
|  |  |  | age ≥ 60 | 0.012 | 1.152 (1.032–1.287) | 3.2×10^-3^ | 1.187(1.059-1.330) |
| rs459193 | *ANKRD55* | G | age ≥ 40 | 0.141 | 1.060 (0.981–1.145) | 0.159 | 1.059(0.978-1.147) |
|  |  |  | age ≥ 50 | 0.133 | 1.067 (0.980–1.162) | 0.119 | 1.070(0.983-1.166) |
|  |  |  | age ≥ 60 | 0.019 | 1.136 (1.021–1.263) | 9.2×10^-3^ | 1.156(1.037-1.289) |
| rs10401969 | *CILP2* | C | age ≥ 40 | 0.983 | 0.999 (0.880–1.134) | 0.974 | 1.002 (0.879-1.142) |
|  |  |  | age ≥ 50 | 0.614 | 0.965 (0.841–1.108) | 0.556 | 0.959(0.835-1.102) |
|  |  |  | age ≥ 60 | 0.948 | 0.994 (0.836–1.183) | 0.798 | 0.977(0.817-1.168) |
| rs12970134 | *MC4R* | A | age ≥ 40 | 0.771 | 1.015 (0.916–1.126) | 0.951 | 0.997(0.896-1.109) |
|  |  |  | age ≥ 50 | 0.767 | 0.983 (0.879–1.100) | 0.599 | 0.970(0.866-1.086) |
|  |  |  | age ≥ 60 | 0.923 | 0.993 (0.863–1.143) | 0.724 | 0.974(0.843-1.126) |
| rs7202877 | *BCAR1* | T | age ≥ 40 | 0.594 | 1.026 (0.934–1.126) | 0.518 | 1.032(0.938-1.136) |
|  |  |  | age ≥ 50 | 0.729 | 1.018 (0.919–1.128) | 0.6 | 1.028(0.927-1.140) |
|  |  |  | age ≥ 60 | 0.744 | 1.022 (0.899–1.161) | 0.673 | 1.029(0.902-1.174) |
| rs11063069 | *CCND2* | G | age ≥ 40 | 0.721 | 1.045 (0.821–1.331) | 0.559 | 1.077(0.840-1.380) |
|  |  |  | age ≥ 50 | 0.878 | 1.021 (0.784–1.329) | 0.873 | 1.022(0.783-1.333) |
|  |  |  | age ≥ 60 | 0.157 | 1.301 (0.903–1.875) | 0.182 | 1.291(0.887-1.878) |
| rs8108269 | *GIPR* | G | age ≥ 40 | 0.53 | 1.026 (0.947–1.112) | 0.428 | 1.034(0.952-1.124) |
|  |  |  | age ≥ 50 | 0.675 | 1.019 (0.933–1.113) | 0.615 | 1.023(0.936-1.118) |
|  |  |  | age ≥ 60 | 0.845 | 1.011 (0.906–1.129) | 0.815 | 1.014(0.905-1.135) |
| rs8090011 | *LAMA1* | G | age ≥ 40 | 0.944 | 1.003 (0.921–1.092) | 0.769 | 1.013(0.928-1.107) |
|  |  |  | age ≥ 50 | 0.757 | 0.985 (0.897–1.082) | 0.863 | 0.992(0.902-1.090) |
|  |  |  | age ≥ 60 | 0.594 | 1.032 (0.919–1.159) | 0.709 | 1.023(0.908-1.152) |
| GRS-7 |  |  | age ≥ 40 | 1.4×10^-3^ | 1.060(1.023-1.099) | 1.1×10^-3^ | 1.063(1.025-1.103) |
|  |  |  | age ≥ 50 | 1.3×10^-3^ | 1.067(1.025-1.109) | 8.5×10^-4^ | 1.070(1.028-1.113) |
|  |  |  | age ≥ 60 | 3.4×10^-6^ | 1.124(1.070-1.180) | 9.4×10^-7^ | 1.135(1.079-1.194) |

Results of logistic regression analysis using all type 2 diabetes participants (n=4,280) are shown

^a^ Information in the original report is shown

^b^ Risk allele reported in the previous reports

^c^ Adjusted for age, sex and BMI

^d^ GRS-7 was calculated according to the number of risk allele of the 7 SNPs, and the individuals having complete genotype data for the 7 SNPs were used for the analysis (age ≥ 40, n = 5,498, age ≥ 50, n = 5,118, age ≥ 60 n = 4,545) adjusted for age, sex and BMI
